# Educational attainment reduces the risk of suicide attempt among individuals with and without psychiatric disorders independent of cognition: a bidirectional and multivariable Mendelian randomization study with more than 815,000 participants

**DOI:** 10.1038/s41398-020-01047-2

**Published:** 2020-11-09

**Authors:** Daniel B. Rosoff, Zachary A. Kaminsky, Andrew M. McIntosh, George Davey Smith, Falk W. Lohoff

**Affiliations:** 1 Section on Clinical Genomics and Experimental Therapeutics, National Institute on Alcohol Abuse and Alcoholism, National Institutes of Health, Bethesda, MD USA; 2 Royal’s Institute of Mental Health Research, University of Ottawa, Ottawa, ON Canada; 3 Division of Psychiatry, Royal Edinburgh Hospital, University of Edinburgh, Edinburgh, UK; 4 MRC Integrative Epidemiology Unit, University of Bristol, Bristol, UK

**Keywords:** Depression, Diagnostic markers

## Abstract

Rates of suicidal behavior are increasing in the United States and identifying causal risk factors continues to be a public health priority. Observational literature has shown that educational attainment (EA) and cognitive performance (CP) influence suicide attempt risk; however, the causal nature of these relationships is unknown. Using summary statistics from genome-wide association studies (GWAS) of EA, CP, and suicide attempt risk with > 815,000 combined white participants of European ancestry, we performed multivariable Mendelian randomization (MR) to disentangle the effects of EA and CP on attempted suicide. In single-variable MR (SVMR), EA and CP appeared to reduce suicide attempt risk (EA odds ratio (OR) per standard deviation (SD) increase in EA (4.2 years), 0.524, 95% CI, 0.412–0.666, *P* = 1.07 × 10^−7^; CP OR per SD increase in standardized score, 0.714, 95% CI, 0.577–0.885, *P* = 0.002). Conversely, bidirectional analyses found no effect of a suicide attempt on EA or CP. Using various multivariable MR (MVMR) models, EA seems to be the predominant risk factor for suicide attempt risk with the independent effect (OR, 0.342, 95% CI, 0.206–0.568, *P* = 1.61 × 10^−4^), while CP had no effect (OR, 1.182, 95% CI, 0.842–1.659, *P* = 0.333). In additional MVMR analyses accounting simultaneously for potential behavioral and psychiatric mediators (tobacco smoking; alcohol consumption; and self-reported nerves, tension, anxiety, or depression), the effect of EA was little changed (OR, 0.541, 95% CI, 0.421–0.696, *P* = 3.33 × 10^−6^). Consistency of results across complementary MR methods accommodating different assumptions about genetic pleiotropy strengthened causal inference. Our results show that even after accounting for psychiatric disorders and behavioral mediators, EA, but not CP, may causally influence suicide attempt risk among white individuals of European ancestry, which could have important implications for health policy and programs aimed at reducing the increasing rates of suicide. Future work is necessary to examine the EA–suicide relationship populations of different ethnicities.

## Introduction

Suicide is a leading cause of death with ~800,000 deaths per year worldwide^1^. While suicide rates vary by country, the age-adjusted suicide rate in the United States has increased 33% from 1999 to 2017 with more than 120 Americans dying by suicide every day^2^. With an estimated 2.1 million discharges for self-inflicted injuries from emergency departments and acute care hospitals reported in the US in 2013 alone^3^ and $50.8 billion in medical expenditures and lost productivity^4^, suicidal behavior is a major public health and economic burden^5,6^. Given the incalculable emotional and psychological costs, the true public health burden of suicidality is difficult to estimate^6^; however, these statistics highlight the importance of identifying causal risk factors for developing effective suicide reduction and prevention strategies^7^.

The relationship between mental health, educational attainment (EA), and cognitive performance (CP) are well documented^8,9^ with up to 54% of college students acknowledging suicidal ideation at some point during college^10^, and a recent longitudinal study showing that a history of suicide planning and attempt(s) prior to matriculation was associated with decreased college academic performance^11^. In addition, observational literature has shown that low EA may be a major risk factor for suicidal behavior throughout the life course^12–15^, which may contribute to the disparity in mortality across socioeconomic strata^16^. However, this relationship between EA and suicide behavior is complex and potentially confounded by CP, which is strongly associated with EA^17,18^ and also linked to suicidal behavior^19^. Like the EA–suicide behavior relationship, there is evidence suggesting a bidirectional association^20,21^.

However, caution is needed when inferring causality from multivariable-adjusted regression in observational data^22–24^, and observational studies are subject to reverse causation and residual confounding^23,25^ not only from correlated factors like CP and other socioeconomic status indices and cultural factors, but also from psychiatric symptoms and disorders, and potential behavioral mediators, also shown to affect suicidal behavior^26,27^. While randomized control trials (RCTs) are the “gold standard” of causal inference^28^, it is impossible (unethical) to construct an RCT examining the effects of either EA or CP on suicidal behavior. Therefore, it remains to be elucidated whether low EA and/or low CP are causes or consequences of suicidal behavior.

Mendelian randomization (MR) is a genetic epidemiology method that uses randomly inherited genetic variants as instrumental variables to assess possible causal relationships between environmental exposures (e.g., EA, CP), and outcomes (e.g., suicidal behavior). As genetic variants are not modifiable by confounders, MR is analogous to RCTs, except randomization occurs at meiosis^29^. If the genetically predicted values of the environmental exposure are associated with the outcome, then causal inference may be drawn from their association^23,30^, and thus MR is an important analytical strategy when RCTs are impractical or unethical^23^.

The pathways to suicidal behavior are complex and involve the dynamic interaction of psychological, genetic, social, and cultural factors^31^, and while the precise genetic basis of suicidal behavior remains largely unknown^31^, studies have shown that the genetic basis of suicidal behavior is polygenic, heritable and, in part, distinct from that of psychiatric disorders^32,33^, while recent sufficiently powered genome-wide association studies (GWASs) have identified the first conventional genome-wide significant (GWS) (*P* < 5 ×10^−8^) loci associated with suicide attempts in both the general population^33^ and in a psychiatric cohort^32^. There are also a growing number of MR studies using genetic data to investigate the relationship between suicide and modifiable risk factors, such as smoking^34^.

EA and CP are strongly influenced by social, cultural, environmental factors^35,36^; however, they are also polygenic and heritable^37,38^. Further, the genetic components explain a non-trivial percentage of their variance with a recent GWAS in 1.1 million participants found polygenic scores constructed to explain between 11–13% and 7–10% of the variance in EA and CP, respectively^37^. EA is also one of the most widely studied exposures in the MR field with evidence for the causal effects of EA on many behaviors and health outcomes, including alcohol consumption patterns and alcohol dependence (AD)^39^, smoking^40,41^, physical activity^42^, and cardiovascular disease^43–45^.

These conventional MR studies suggest that increasing years of education would help address health disparities, but failing to account for CP, which could be potentially driving the observed protective effect of increased EA if not accounted for in the analyses has important for implications for the formulation and evaluation of targeted prevention programs aimed at increasing EA to reduce morbidity and mortality associated with these outcomes and would be important for strategies aimed at reducing suicide. Multivariable MR (MVMR) is a recently developed method that allows for simultaneous assessment of separate but correlated exposures^41,46^ by incorporating genetic variants from each risk factor into the same model^42^. MVMR has been recently employed to disentangle the independent effect for each risk factor for a range of health outcomes: e.g., Richardson et al.^47^ recently used an MVMR framework to compare the causal roles of lipids and apolipoproteins with coronary heart disease found that the effects of low-density lipoprotein cholesterol (LDL-C) were attenuated in MVMR models accounting for other lipids and lipoproteins. Similarly, MVMR has been used to evaluate the total and direct effects of EA, and CP with many health and behavioral outcomes including smoking^48^, alcohol consumption, physical activity, and body mass index (BMI)^42^.

In this study, we used the largest publicly available GWASs on EA (*N* ≤ 766,345)^37^, CP (*N* ≤ 257,828)^37^, hospital-based records of either a primary or secondary diagnosis of a suicide attempt (*N* ≤ 50,264)^49^ to comprehensively examine the relationship between EA, CP, and suicide attempts. We first performed a conventional bidirectional MR analysis to identify the direction of the relationship(s). We then leveraged recently developed MVMR methods to identify whether EA or CP to identify the direct causal effect of one exposure controlling for the other (i.e., the effect of EA holding CP constant). Finally, given the strong genetic correlation of EA and CP with markers of socioeconomic status (SES)^50,51^, and, further, possible confounding from comorbid mental disorders (major depressive disorder (MDD), schizophrenia, and bipolar disorder (BD)), psychiatric symptoms (feeling tense, anxious, or depressed), and potential behavioral mediators (tobacco smoking and alcohol consumption) we performed additional MVMR analyses incorporating household income^52^, MDD, schizophrenia, BD, alcohol consumption^53^, past or current smoking status^52^, self-reported visits to a general practitioner (GP) or psychiatrist for nerves, anxiety, tension or depression (*N* ≤ 460,702)^52^ to evaluate the robustness of the main EA–suicide analysis.

## Methods

### Study design and data sources

**Fig. 1:**
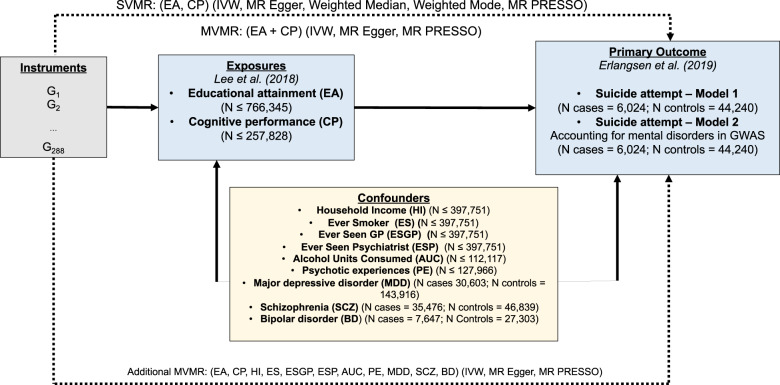
Study design schematic. Dashed lines indicate Mendelian randomization (MR) analyses of SVMR and MVMR exposure instruments on the outcome suicide attempt risk; MR methods/sensitivity analyses in parentheses. *G*_i_ instrument variants, EA educational attainment, CP cognitive performance, Income average household income before tax, MR Mendelian randomization, MVMR multivariable MR, SVMR single-variable MR, IVW inverse-variance weighted MR, MR-PRESSO Mendelian Randomization Pleiotropy Residual Sum, and Outlier, SNP single-nucleotide polymorphism. Instruments used were the genome-wide significant (*P* < 5 × 10^−8^) single-nucleotide polymorphisms (SNPs) extracted from each of the educational attainment (EA) and cognitive performance (CP) GWASs, as well as from each of the confounders, for the SVMR analyses, combined for the MVMR analyses, that were independent at a linkage disequilibrium (LD) *r*^2^ < 0.001, with clumping distance set at 10,000 kb, and found in each of the outcome suicide attempt risk GWASs.

Figure 1 displays the overall design of the study for the two-sample SVMR and MVMR analyses of the effect of EA and CP on the risk of a suicide attempt—adjusting for total household income (before taxes), alcohol consumption, past or current smoking status, and self-reported visits to a general practitioner or psychiatrist for nerves, anxiety, tension or depression. We used publicly available summary statistics from the Social Science Genetic Association Consortium (SSGAC), the Lundbeck Foundation Initiative for Integrative Psychiatric Research (iPSYCH), and the Medical Research Center-Integrative Epidemiology Center, UK Bristol (MRC-IEU) UK Biobank GWAS Pipeline (Supplementary Table 1; web links for downloading data provided). All studies have existing ethical permissions from their respective institutional review boards and include participant informed consent and included rigorous quality control. As all analyses herein are based on publicly available summary data, no ethical approval from an institutional review board was required.

### Instruments

We extracted instrument SNPs for EA from the recent SSGAC GWAS meta-analysis of 70 separate GWASs from a total of 766,345 individuals of European ancestry including 442,183 from the UK Biobank prospective cohort study collected across the United Kingdom from 2006 to 2010^37^. Using the standard heuristic significance threshold for these dimensional MR studies to make the work manageable, we included all SNPs associated at genome-wide significance (GWS) (*P* < 5 × 10^−8^) with the phenotype “educational attainment” (EA); due to educational systems differences across cohorts, EA had been constructed for the GWAS meta-analysis from an imputed years-of-education equivalent for each International Standard Classification of Education category mapped from each cohort’s survey measures. Across all cohorts (excluding the 23andMe cohort due to institutional restrictions), the sample-size weighted mean of EA is 16.8 years of schooling (standard deviation (s.d.) 4.2 years). Genetic association results are presented in s.d. units. We pruned the resultant list of GWS SNPs to exclude all SNPs with a pairwise linkage disequilibrium (LD) *r*^2^ > 0.001 (Supplementary Table 2).

We extracted SNPs for CP from the companion SSGAC GWAS meta-analysis of the Cognitive Genomics Consortium (COGENT) GWAS on general cognitive performance in 35,298 individuals, combined with the GWAS of cognitive performance in 222,543 individuals of the UK Biobank prospective cohort study, both of predominantly European ancestry (sample size *N* = 257,841). We included all SNPs associated with CP at GWS (*P* < 5 × 10^−8^). In the COGENT study, the phenotype was defined as the first principle component of three or more neuropsychological tests, including but not limited to tests assessing digit span, digit symbol coding, phonemic fluency, semantic fluency, trail-making, verbal memory for stories, verbal memory for words, visual memory, vocabulary and word reading (mean = 0.00, s.d. = 0.99); in the UK Biobank, the phenotype was defined as the standardized score (mean = 0.00, s.d. = 1.00) on up to four tests comprised of a 2-min verbal-numerical assessment of fluid intelligence (comprised of thirteen logic and reasoning questions): the genetic correlation between the score and general intelligence among children has been shown in prior studies to be ~0.8. SNP heritability for cognitive performance was found to be 0.209 (standard error (s.e.) = 0.007). LD score regression indicated a genetic overlap between cognitive performance and years of schooling (rG = 0.662, s.e. = 0.011). Genetic association results are presented in s.d. units. We pruned the resultant list of GWS SNPs to exclude all SNPs with a pairwise LD *r*^2^ > .001 (Supplementary Table 3).

To account for income in our MVMR analyses, we extracted SNPs from the MRC-IEU UK Biobank GWAS based on the responses of 397,751 individuals of European ancestry from the UK Biobank prospective cohort study^52^. We included all SNPs associated at GWS (*P* < 5 × 10^−8^) with the PHESANT-derived phenotype (UK Biobank data-field 738) from five categorically ordered responses of the study, with the first category “less than £18,000”, up the fifth category “more than £100,000”, categorizing average total household income before tax (income). We pruned the results to exclude all SNPs with a pairwise LD *r*^2^ > 0.001 (Supplementary Table 4).

To account for alcohol consumption, we extracted SNPs from the 2017 SSGAC GWAS for units of alcohol consumption based on the responses of 112,117 individuals of European ancestry from the UK Biobank prospective cohort study on average weekly alcohol consumption of a range of alcohol drink types, including red wine, white wine, champagne, spirits, beer, and cider^53^. We included all SNPs associated at GWS (*P* < 5 × 10^−8^) with the constructed phenotype and pruned the results to exclude all SNPs with a pairwise LD *r*^2^ > 0.001, leaving 36 independent SNPs (Supplementary Table 5). Mean consumption for respondent females was 10.0 alcohol units per week (standard deviation (s.d.) = 1.8) and for males, 20.8 units per week (s.d. = 19.0), and, overall, 15.1 units per week (s.d. = 16.6): large genetic overlap and genetic overlap between male and female consumption were found (rG = 0.90, s.e.= 0.09, *P* = 16 × 10^−23^). SNP heritability was 0.13 (standard error (s.e.) = 0.01) overall, 0.15 (s.e. = 0.01) for males, and 0.13 (s.e. = 0.01) for females. LD score regression indicated a genetic overlap between alcohol consumption and years of schooling (rG = 0.18, s.e. = 0.03).

We extracted SNPs from the MRC-IEU UK Biobank GWAS to account for ever smoker status based on the responses of 461,066 individuals of European ancestry (280,508 cases; 180,588 controls) again from the UK Biobank prospective cohort study^52^. We included all SNPs associated at GWS (*P* < 5 ×10^−8^) with the PHESANT-derived phenotype (UK Biobank data-field 20160), constructed from responses on current and past tobacco smoking: individuals were classified as “ever smoker” if they responded “most days” or “occasionally” to the current tobacco smoking query or if they responded “most days”, “occasionally”, or “tried once or twice” to the past tobacco smoking query. We pruned the results to exclude all SNPs with a pairwise LD *r*^2^ > 0.001 (Supplementary Table 6).

Similarly, we extracted SNPs from the MRC-IEU UK Biobank GWAS to account for nerves, tension, anxiety or depression, based alternatively upon the responses of 459,560 individuals of European ancestry (158,561 cases; 300,995 controls) to the query about ever having seen a general practitioner for nerves, tension, anxiety or depression, and upon the responses of 460,702 individuals (53,414 cases; 407,288 controls) to the query about ever having seen a psychiatrist for nerves, tension, anxiety or depression, in the UK Biobank prospective cohort study^52^. We included all SNPs associated at GWS (*P* < 5 × 10^−8^) with the PHESANT-derived phenotypes (UK Biobank data-field 2090 and 2100, respectively), pruned to exclude all SNPs with a pairwise LD *r*^2^ > 0.001 (Supplementary Table 7). To account for having a history of any psychotic experiences, we used summary association data from the recent Legge et al.^54^ GWAS in the UK Biobank (6123 cases; 121,843 controls) (Supplementary Table 1). We pruned the results to exclude all SNPs with a pairwise LD *r*^2^ > 0.001 (Supplementary Table 8).

To account for potential confounding by major psychiatric disorders, we included additional analyses using summary association data from three GWAS that did not include the iPSYCH cohort in the analysis—probable MDD (30,603 cases; 143, 916 controls)^55^, schizophrenia (35,476 cases; 46, 839 controls)^56^, and BD (7647 cases; 27,303 controls)^57^ (Supplementary Table 1). We pruned the association data to exclude all SNPs with a pairwise LD *r*^2^ > 0.001 (Supplementary Table 8). The samples were of white, European ancestry, except for the schizophrenia data were of predominantly white, European ancestry, but contained 1866 cases and 3414 controls of East Asian ancestry, too^56^.

For our MVMR analyses combining EA and CP, along with income, alcohol consumption, ever smoker status, and alternatively, ever having seen a general practitioner or psychiatrist for nerves, tension, anxiety, or depression, we included all independent SNPs (LD *r*^2^ < 0.001; 10,000 kb window), which were GWS (*P* < 5 × 10^−8^) in any the GWASs (Supplementary Table 9).

### Suicide attempt outcomes

We extracted summary statistics on the associations of the instruments SNPs with suicide attempt (SA) from the recent iPSYCH GWASs in a sample constructed from a cohort of 1,472,762 singletons born in Denmark between 1981 and 2005 and residing in Denmark on their first birthday, including 57,377 participants diagnosed with one or more mental disorders according to the 10th Revision of International Classification of Diseases (i.e., schizophrenia, bipolar disorders, affective disorders, autism spectrum disorder, anorexia) and, additionally, a non-psychiatric population-based random sample of 30,000 participants drawn also from the cohort^49^. Sample participants were further screened by the Danish Psychiatric Central Research Register and National Registry of Patients for diagnoses of non-fatal suicide attempts; cases also included those with combinations of diagnoses with the main diagnoses recorded as a mental disorder, and a secondary diagnosis recorded as poisoning by drugs or other substances, or injuries to the hand, wrist, and forearm, well-established proxies for suicide attempt^49^. The control group consisted of all persons not diagnosed with one or more suicide attempts with and without diagnosed mental disorders. After quality control, and excluding children, 50,264 persons remained in the sample for the GWAS, of which 6024 (12%) recorded at least one suicide attempt, and 44,240 (88%) did not^49^. For the 6024 cases, 17.9% were aged 15–19 years, 37.2% 20–24 years, 35.6% 25–29 years, and 9.4% 30–34 years; 54.7% of the cases had 1 recorded suicide attempt; 22.4%, 2; 14.8%, 3–4; 6.9%, 5–9; and 2.0%, 10 or more.

The iPSYCH GWAS (model 1) used for the main anlayses did not account for comorbid mental disorders: the GWAS was conducted using logistic regression to calculate the log odds ratio of the risk of suicide attempt with the only gender, years followed, and first ten principal components of genetic ancestry included as covariates. The companion iPSYCH GWAS (model 2) accounted for comorbid mental disorders by including a series of binary covariates for diagnoses of the following: schizophrenia, bipolar disorder, affective disorder, autism spectrum disorders, anorexia, and “any other disorder”. We adopted the “Model 1” and “Model 2” notation for the purposes of this study. Cognizant of the potential for collider bias in GWAS analyses adjusting for environmental covariates since genetic variants may be, in theory, associated with both the primary outcome (here suicide attempt) and the covariates used for an adjustment (i.e., psychiatric disorders)^58^, we used the iPSYCH model 2 solely as a sensitivity analysis used in addition to the MVMR analyses including psychiatric symptoms, alcohol consumption, smoking, MDD, schizophrenia, and bipolar disorder.

### Extraction of instruments from outcome GWASs and harmonization of effect alleles

Of the 318 possible GWS SNPs associated with EA, 270 SNPs were present in both iPSYCH models 1 and 2 suicide attempt (SA) risk GWASs, and 46 SNPs were removed during harmonization for being palindromic with intermediate allele frequency, leaving 224 SNPs for SVMR analysis. Of the 157 possible SNPs associated with CP, 127 SNPs were present in the SA GWASs, and 23 SNPs were removed during harmonization for being palindromic with intermediate allele frequency, leaving 104 SNPs for SVMR analysis. Of the 333 possible independent SNPs of the combined MVMR instrument set, 265–288 SNPs were present in the SA GWASs, depending on the combination of exposures. We calculated the *F*-statistics and the *F*-statistic 95% confidence interval (95% CI) lower bound (in parentheses) for each exposure to assess the strength of each of the instruments present in the SA GWAS, and all exceeded the threshold *F*-statistic of 10 recommended for MR analysis^59^ (EA *F*-statistic 59.8 (95% CI lower bound 58.1); CP 55.2 (52.9); household income: 45.3 (41.9); alcohol consumption 31.8 (28.6); ever smoker status 48.8 (45.8); ever seen a general practitioner for nerves, tension, anxiety or depression 31.0 (27.6); and ever seen a psychiatrist for nerves, tension, anxiety or depression: 26.4 (23.9) (Supplementary Table 10). We were unable to calculate conditional *F*-statistics to assess the strength of our multivariable instruments: SVMR statistical methods recently extended by Sanderson et al.^60^ to two-sample MVMR are appropriate only for non-overlapping exposure summary-level data sources; when overlapping, the requisite pairwise covariances between SNP associations are determinable only using individual-level data.

### Sample independence

Participant overlap in samples used to estimate genetic associations between exposure and outcome in two-sample MR can bias results^46,61^, so we endeavored to minimize overlap to reduce this source of weak instrument bias. In this study, exposure and outcomes were derived from non-overlapping samples. Exposures, however, were, derived from overlapping UKBB-based samples. As regards potential biases arising from participant overlap in samples used to construct the multivariable instrument sets, we are unaware of any tests to assess weak instrument bias arising therefrom, however, unconditional single variable and conditional multivariable *F*-statistics assessing the strength of the instruments may be used to indicate any overall weak instrument bias. We calculated unconditional SVMR instrument *F-*statistics, all of which exceeded the rule-of-thumb *F* > 10, but, as described above, we are not able to calculate conditional MVMR instrument *F-*statistics.

### Statistical analysis

For SVMR, applied to assess the total effects of EA and CP on the risk of suicide attempt, with and without accounting for comorbid mental disorders, and vice versa, we used inverse-variance weighted MR (MR IVW) (single-variable weighted linear regression) along with the complementary MR Egger, weighted median and weighted mode methods, to assess the evidence of the causal effects of EA and CP on the risk of suicide attempt, so as to detect the sensitivity of the results to different patterns of violations of IV assumptions: consistency of results across methods strengthens an inference of causality^62^. MR IVW is generally regarded as the main method: in the absence of pleiotropy and assuming the instruments are valid, MR IVW returns unbiased estimates of a causal effect are returned so long as horizontal pleiotropy is balanced^62,63^. MR Egger extends MR IVW by not setting the intercept to zero, thus allowing the net-horizontal pleiotropic effect across all SNPs to be unbalanced or directional (i.e., some SNPs could be acting on the outcome through a pathway other than through the exposure)^63,64^. MR Egger returns unbiased causal effect estimates even if the assumption of no horizontal pleiotropy is violated for all SNPs, but the estimates are less precise than MR IVW. Weighted median MR uses the median effect of all available SNPs so that only half of the SNPs need to be valid instruments (i.e., no horizontal pleiotropy, no association with confounders, and robust association with the exposure) to return an unbiased causal effect estimate. Stronger SNPs contribute more towards the causal estimate, with the contribution of each SNP weighted by the inverse variance of its association with the outcome^65^. Weighted mode-based MR clusters the SNPs into groups based on the similarity of causal effects and returns the causal effect estimate based on the cluster that has the largest number of SNPs: unbiased causal effects so long as the SNPs within the largest cluster are valid instruments. Weighted mode MR weights each SNP’s contribution to the clustering by the inverse variance of its outcome effect. Assuming the most common causal effect is consistent, the estimated causal effects would be unbiased even if all other instruments are invalid^66^.

For MVMR, performed to assess the independent direct effects of EA and CP on the risk of suicide attempt, with and without accounting for comorbid mental disorders and other potential confounders, including household income, alcohol consumption, ever smoker status, and self-reported nerves, tension, anxiety or depression, we used the extension developed by Burgess et al.^63^ of the IVW MR method, performing multivariable weighted linear regression (variants uncorrelated, random effect model) with the intercept term set to zero, and, additionally, the MVMR extension of the MR Egger method to correct for both measured and unmeasured pleiotropy^67^.

### Sensitivity analyses and diagnostics

We used the iPsych model 2 (GWAS on suicide attempts accounting for psychiatric diagnoses) as a primary sensitivity analysis to the main model 1 findings. To evaluate heterogeneity in genetic instrument effects, indicating potential violations of the instrumental variable (IV) assumptions, we used MR Egger intercept test^68^, the Cochran *Q* heterogeneity test^69^, and the MR pleiotropy residual sum and outlier (MR-PRESSO) test^70^. An MR Egger regression intercept is generally interpreted as the average pleiotropic effect across all instruments: MR Egger regression thus provides a test for average pleiotropy, and further has been extended to correct for both measured and unmeasured pleiotropy in MVMR^67^. The Cochran *Q* test used to identify outliers in regression analysis generally has been applied in MR to detect average pleiotropy: pleiotropy can induce heterogeneity of individual ratio estimates^70^. MR-PRESSO detects pleiotropic bias in MR caused by a violation of the exclusion restriction IV assumption; extending the principal of the Cochran *Q* test, MR-PRESSO provides a global test to detect pleiotropic bias and identify sources of the bias (so-called outlier SNPs), and further has been extended to detect pleiotropic bias also in MVMR^70^. We used the MR-PRESSO global test to identify outlier SNPs; removing the outlier SNPs, we reran the MR and retested so as to determine whether removing outliers resolved the detected heterogeneity. The MVMR models accounting for psychiatric symptoms (psychotic experiences, feeling anxious and/or depressed), behaviors (alcohol consumption and smoking), and psychiatric disorders (MDD, schizophrenia, and BD) were also used as additional sensitivity analyses to evaluating the robustness of the main EA–suicide findings

### Interpretation of findings

While we caution against interpreting the study findings based solely on the basis of a dichotomous *P*-value threshold^71^, we used a two-sided alpha of 0.025 (based upon testing EA and CP) to dichotomize “significant” or not.

## Results

**Fig. 2:**
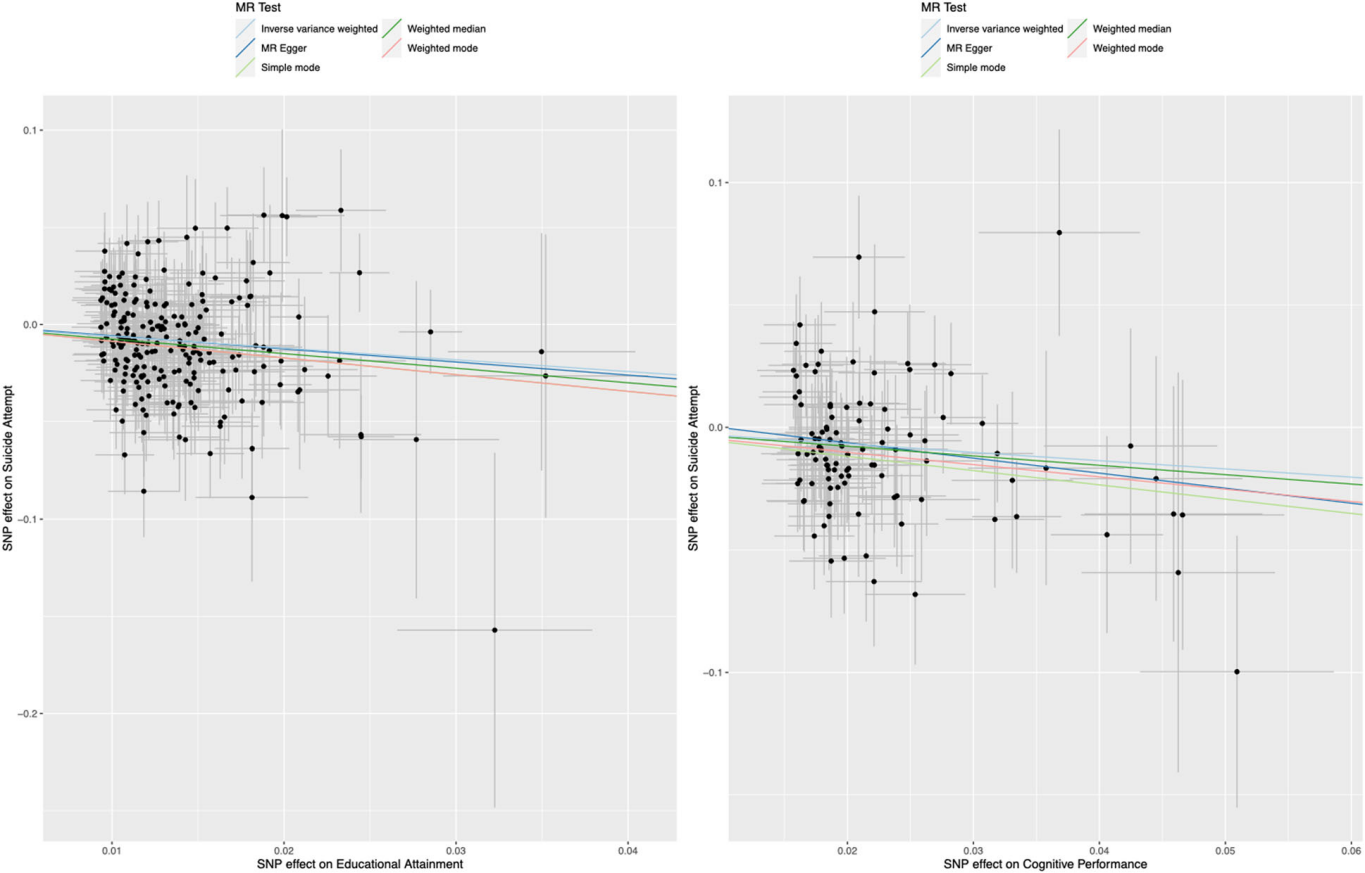
Single-variable Mendelian randomization analysis for (LEFT) educational attainment and (RIGHT) cognitive performance on risk of suicide attempts (not accounting for comorbid mental disorders). MR Mendelian randomization, SNP single-nucleotide polymorphism.

We generally looked for those estimates (1) substantially agreeing in direction and magnitude across complementary MR methods, (2) exceeding nominal significance in MR IVW, (3) not indicating bias from horizontal pleiotropy (MR-PRESSO global *P* > 0.01; and/or also MR Egger intercept *P* > 0.01), and, for SVMR, (4) indicating true causal effect directionality (Steiger directionality test *P* < 0.01). Complete MR results with test statistics are presented in Supplementary Tables 9–12. MR-PRESSO outlier corrected results are presented in Tables 1, 2 and Fig. 2.

**Table 1:** Single-variable and multivariable inverse-variance weighted mendelian randomization associations between educational attainment and cognitive performance on risk of suicide attempt in individuals with and without mental disorders.

	Outcome									
	Suicide attempt: model 1					Suicide attempt: model 2				
Exposures	N SNPS	OR	OR LCI	OR UCI	*P*-value	N SNPS	OR	OR LCI	OR UCI	*P*-value
*SVMR*										
Educational attainment	223	0.524	0.412	0.666	1.07E−07	224	0.687	0.540	0.874	2.20E−03
Cognitive performance	104	0.714	0.577	0.885	2.02E−03	104	0.794	0.636	0.990	4.01E−02
Household income	41	0.591	0.389	0.901	1.41E−02	41	0.721	0.480	1.081	1.13E−01
*MVMR*										
Educational attainment	288	0.450	0.314	0.644	1.00E−04	288	0.556	0.388	0.796	2.00E−03
Cognitive performance	288	1.044	0.764	1.426	7.86E−01	288	1.081	0.789	1.482	6.29E−01
*MVMR: adjusting for Income*										
Educational attainment	282	0.342	0.206	0.568	1.61E−04	283	0.437	0.258	0.739	2.00E−03
Cognitive performance	282	1.182	0.842	1.659	3.33E−01	283	1.143	0.803	1.627	4.57E−01
Household income	282	1.101	0.627	1.932	7.38E−01	283	1.225	0.683	2.197	4.96E−01

Results are presented as odds ratios (OR) with 95% confidence intervals for the effect of a unit standard deviation increase in educational attainment (years of schooling: mean = 15.1, s.d.=4.2 years), a unit standard deviation increase in standardized cognitive performance score (mean 0.00, s.d. = 0.99–1.00), and categorical increase in average annual household income before tax, on the risk of suicide attempt (hospital recorded non-fatal suicide attempt, including secondary diagnoses of poisoning by drugs or other substances, or injuries to the hand, wrist, and forearm). Model 1 was based upon iPSYCH Suicide Attempt Risk GWAS not accounting for comorbid mental disorders (*N* = 50,260); model 2 was based upon iPSYCH Suicide Attempt Risk GWAS accounting for diagnosed comorbid mental disorders in the same cohort sample (*N* = 50,260): schizophrenia, bipolar disorder, affective disorders, autism spectrum disorder, anorexia, and “any other disorder”. (1) SVMR results show effects of exposures on outcomes analyzed separately: the estimates are considered to be the total effect (direct plus indirect effect) of the exposure on the outcome; (2) MVMR results show effects of EA and CP analyzed simultaneously: the estimates are interpreted as the direct effect of the exposure on the outcome, independent of the effect of the other exposure; (3) MVMR adjusting for Average Household Income (Before Tax) showing effects of EA, CP and AHI analyzed simultaneously. All results shown are pruned of variants identified as outliers by the MR-PRESSO test (MR-PRESSO *P* < 0.10). Cochran *Q* tests did not indicate heterogeneity and MR Egger intercept test did not indicate pleiotropy for any model. See Supplementary Tables 10–12 for full results.

*SVMR* single-variable Mendelian randomization, *MVMR* multivariable Mendelian randomization, *N* number, *SNPs* single-nucleotide polymorphisms, *OR* odds ratio, *OR LCI* 95% confidence interval lower bound, *OR UCI* 95% confidence interval upper bound.

**Table 2:** Multivariable inverse-variance weighted mendelian randomization association between educational attainment and risk of suicide attempt in individuals with and without mental disorders, adjusted for tobacco smoking behavior, alcohol consumption behaviors, and whether ever seen a general practitioner or psychiatrist for nerves, tension, anxiety or depression.

	Outcome									
	Suicide attempt: model 1					Suicide attempt: model 2				
Exposures	N SNPS	OR	OR LCI	OR UCI	*P*-value	N SNPS	OR	OR LCI	OR UCI	*P*-value
*SVMR*										
Educational attainment	223	0.524	0.412	0.666	1.07E−07	224	0.687	0.540	0.874	2.20E−03
Alcohol consumption	32	1.041	0.591	1.837	8.90E−01	33	1.113	0.602	2.058	7.32E−01
Ever smoker	54	7.017	2.660	18.602	8.26E−05	54	3.899	1.561	9.734	3.56E−03
Ever seen a GP	28	12.315	3.462	44.099	1.05E−04	NA				
Ever seen a psychiatrist	47	28.979	5.158	164.237	1.32E−04	NA				
*MVMR: Adjusting for alcohol consumption and ever smoker status*										
Educational attainment	262	0.481	0.385	0.600	5.02E−10	265	0.624	0.495	0.787	8.01E−05
Alcohol consumption	262	0.757	0.404	1.418	3.85E−01	265	0.781	0.407	1.497	4.57E−01
*MVMR: adjusting ever smoker status*										
Educational attainment	278	0.511	0.406	0.643	2.46E−08	279	0.633	0.503	0.796	1.17E−04
Ever smoker	278	5.778	2.143	15.576	1.00E−03	279	4.225	1.576	11.323	4.00E−03
*MVMR: Adjusting for alcohol consumption and ever smoker status*										
Educational attainment	263	0.500	0.389	0.642	1.42E−07	276	0.640	0.502	0.816	4.02E−04
Alcohol consumption	263	0.766	0.384	1.526	0.448	276	0.654	0.334	1.282	2.17E−01
Ever smoker	263	4.141	1.412	12.146	0.01	276	3.770	1.339	10.611	1.20E−02
*MVMR: adjusting for alcohol consumption, ever smoker status, and ever seen GP*										
Educational attainment	279	0.545	0.425	0.699	3.33E−06	NA				
Alcohol consumption	279	0.757	0.386	1.482	4.16E−01	NA				
Ever smoker	279	4.389	1.433	13.439	1.00E−02	NA				
Ever seen a GP	279	2.971	0.687	12.847	1.45E−01	NA				
*MVMR: adjusting for alcohol consumption, ever smoker status, and ever seen psychiatrist*										
Educational attainment	279	0.541	0.421	0.696	2.84E−06	NA				
Alcohol consumption	279	0.768	0.390	1.513	4.46E−01	NA				
Ever smoker	279	5.155	1.707	15.571	4.00E−03	NA				
Ever seen a psychiatrist	279	8.882	0.518	152.323	1.32E−01	NA				

Results are presented as odds ratios (OR) with 95% confidence intervals for the effect of a unit standard deviation increase in educational attainment (years of schooling: mean = 16.8, s.d.=4.2 years), a unit increase in alcohol units consumed weekly (mean 15.1, s.d. = 16.6), a unit increase in the log odds of ever smoking (tobacco), a unit increase in the log odds of every having seen a general practitioner (GP) or psychiatrist, respectively, for nerves, tension, anxiety, or depression on the risk of suicide attempt (hospital recorded non-fatal suicide attempt, including secondary diagnoses of poisoning by drugs or other substances, or injuries to hand, wrist, and forearm). Model 1 was based upon iPSYCH Suicide Attempt Risk GWAS not accounting for diagnosed comorbid mental disorders (*N* = 50,260); model 2 was based upon iPSYCH Suicide Attempt Risk GWAS accounting for diagnosed comorbid mental disorders in same cohort sample (*N* = 50,260): schizophrenia, bipolar disorder, affective disorders, autism spectrum disorder, anorexia, and “any other disorder”. (1) SVMR results show effects of additional exposures on outcomes analyzed separately: the estimates are considered to be the total effect (direct plus indirect effect) of the exposure on the outcome; (2) MVMR results adjusting for additional exposures show effects of EA analyzed simultaneously with additional exposure (and combinations of exposures): the estimates are interpreted as the direct effect of the exposure on the outcome, independent of the effect of the other exposure. All results shown are pruned of variants identified as outliers by the MR-PRESSO test (MR-PRESSO *P* < 0.10). Cochran *Q* tests did not indicate heterogeneity (except as otherwise noted in the text) and MR Egger intercept test did not indicate pleiotropy for any model. See Supplementary Tables 10–12 for full results.

*SVMR* single-variable Mendelian randomization, *MVMR* multivariable Mendelian randomization, *N* number, *SNPs* single-nucleotide polymorphisms, *OR* odds ratio, *OR LCI* 95% confidence interval lower bound, *OR UCI* 95% confidence interval upper bound.

### Total effects of educational attainment and cognitive performance

Using SVMR to assess the total effects of each exposure on our two models of the risk of suicide attempt (models 1 and 2; model 2 used summary statistics from the SA GWAS accounting for comorbid mental disorders). We found genetic variants associated with increased EA, not controlling/adjusting for CP, associated with decreased risk of suicide attempt (OR per unit standard deviation increase in EA, 0.524, 95% CI, 0.412–0.666, *P* = 1.07 × 10^−7^), and genetic variants associated with increased CP, not controlling/adjusting for EA, also associated with decreased risk of suicide attempt (OR per unit standard deviation increase in standardized score, 0.714, 95% CI, 0.577–0.885, *P* = 0.002). Accounting for comorbid mental disorders using the SA model 2 GWAS, we found associations of greater magnitude than in model 1 but less precision for both EA (model 2 OR, 0.687, 95% CI, 0.577–0.885, *P* = 0.002) and CP (model 2 OR, 0.794, 95% CI, 0.636–0.990, *P* = 0.040) (Table 1, lines 1, 2). Conversely, we found no evidence of bidirectionality: genetic variants associated with risk of suicide attempt (with and without accounting for comorbid psychiatric disorders) were not found to be associated with EA (OR 0.996, 95% CI, 0.984–1.007, *P* = 0.463) or CP (OR 1.003, 95% CI 0.983–1.023, *P* = 0.758) (Supplementary Table 11).

### Independent effects of educational attainment and cognitive performance

Using MVMR to simultaneously assess the direct/ independent effects of each of EA and CP by controlling for the other, we found increased EA still associated, after controlling/adjusting for CP, with decreased risk of suicide attempt, with a greater magnitude for the point estimate of the direct effect but within the 95% confidence interval of the total effect estimate (OR, 0.450, 95% CI, 0.314–0.644, *P* < 1 × 10^−4^). Conversely, controlling/adjusting for EA, we found CP no longer associated with the risk of suicide attempt (OR, 1.044, 95% CI, 0.764–1.426, *P* = 0.786). Adjusting further for income, we found increased EA still associated with decreased risk of suicide attempt: the direct effect was greater (lower OR), and no longer within the 95% confidence interval of the total effect estimate (OR, 0.342, 95% CI, 0.206–0.568, *P* = 1.61 × 10^−4^); we found CP still no longer associated directly with the risk of suicide attempt (OR, 1.182, 95% CI, 0.842–1.659, *P* = 0.333) (Fig. 1; Table 1, lines 3–4, compared to lines 1–2; also Supplementary Table 12).

Accounting for comorbid mental disorders using the SA model 2 GWAS, as well as controlling for income via MVMR, we found increased EA still significantly associated with decreased risk of suicide attempt, but to a lesser extent and precision than found using model 1 (model 2 OR, 0.556, 95% CI, 0.388–0.796, *P* < 2 × 10^−3^). CP, when adjusting for EA, was still no longer significant (model 2 OR, 1.143, 95% CI, 0.803–1.627, *P* = 0.475) (Table 1, lines 6–7 and Supplementary Table 12).

Controlling for alcohol consumption and smoking behaviors, we found the effects of genetic variants associated with increased EA to be robust to the inclusion of genetic variants for these potential confounders (OR, 0.500, 95% CI, 0.389–0.642, *P* = 1.42 × 10^−7^), albeit with residual heterogeneity (Table 2, also Supplementary Table 12). Additionally, accounting for comorbid mental disorders using the SA model 2 GWAS, as well as alcohol consumption and smoking behaviors, we found increased EA still significantly associated with decreased suicide attempt again to a lesser extent and precision than found using model 1 (model 2 OR, 0.640, 95% CI, 0.502–0.816, *P* < 4.02 × 10^−4^).

Even controlling for having seen a GP or psychiatrist for symptoms of “nerves, tension, anxiety, or depression,” in addition to alcohol consumption and smoking behaviors (as opposed to using the SA model 2 GWAS, which included incidence of comorbid severe mental disorders among the participants in the GWAS regression), we again found the effects of genetic variants associated with increased EA to be broadly consistent and robust to the inclusion of these potential confounders (OR, 0.545, 95% CI, 0.425–0.699, *P* = 3.33 × 10^−6^, controlling for ever having seen a GP for nerves, tension, anxiety or depression; OR, 0.541, 95% CI, 0.421–0.696, *P* = 2.84 × 10^−6^, controlling for ever having seen a psychiatrist, etc.) (Table 2 and Supplementary Table 12). Further, in supplemental analysis, controlling for any psychotic experiences (PE), MDD, schizophrenia, and BD, genetic variants associated with increased EA were similarly found to be broadly consistent and robust (any psychotic experiences, OR, 0.468, 95% CI, 0.376–0.583, *P* = 1.14 × 10^−11^, controlling for PE; OR, 0.746, 95% CI, 0.578–0.963, *P* = 0.024, *P* = 1.66 × 10^−3^, MDD; OR, 0.631, 95% CI, 0.492–0.811, schizophrenia; and OR, 0.685, 95% CI, 0.541–0.867 *P* = 3.18 × 10^−4^, BD) (Supplementary Table 12).

As regards the potential confounders, we found associations of genetic variants associated with the incidence of smoking behaviors and nerves, tension, anxiety or depression on risk of suicide attempt, when analyzed separately in SVMR (Table 2 and Supplementary Table 11). When analyzed in combination in MVMR, only the variants associated with the incidence of smoking behaviors remained significantly associated with risk of suicide attempt (OR, 5.155 per unit increase in log odds of ever smoking, 95% CI, 1.707–15.571, *P* = 4.00 × 10^−3^).

The MR-PRESSO global tests and Cochran *Q* tests did not indicate heterogeneity, nor did the MR Egger intercepts indicate directional pleiotropy in any of the analyses, after outliers identified by MR-PRESSO were removed as sources of potential pleiotropy (Supplementary Tables 10–12).

## Discussion

In the current study, we use various MR methods to assess the relationship between EA, CP, and risk of a suicide attempt. Our findings highlight an important possible public health benefit of increasing EA: reducing the risk of suicidal behavior. Using multivariable MR, accounting for both CP and income, we found an additional 4.2 years of EA reduces the risk for having attempted suicide suggesting a causal pathway between EA and suicide attempt that is independent of CP, or socioeconomic status, as measured by income. Further, this EA–suicide relationship remained in additional analyses accounting for potential behavioral and psychiatric mediators (tobacco smoking, alcohol consumption, psychotic experiences, mood and anxiety symptoms, etc.), and psychiatric disorders—MDD, schizophrenia, and BD. Given the robust association between suicide attempts and later completed suicide with a recent 25-year longitudinal study estimating ~5% of suicide attempters^72^, these findings highlight an important modifiable factor to mitigate the mortality and morbidity due to suicidal behavior.

Notably, the inverse relationship between CP and risk of suicide attempt found in our SVMR analysis no longer remained after accounting for EA: the relationship appears to be mediated through EA. Conversely, we found in a bidirectional analysis that the genetic liability for suicide attempts affected neither the EA nor CP outcomes, suggesting that previous conventional analyses identifying such effects were due to confounding or reverse causation. Consistent estimates of the effect of EA across complementary MR methods used to assess sensitivity to of these estimates to changes in the underlying IV assumptions strengthens our inference of causality.

This study contributes to the increasing number of MVMR studies reporting an effect of EA on health outcomes after accounting for CP^42,45,48,73^, which, given the disproportionate disparity in suicide rates between individuals with a high school education or less compared to college graduates^14,74^, suggests a contribution to the substantial health inequalities observed associated with differences in EA^75^. Other study designs using innovative approaches have also found evidence for the causal effects of education on health outcomes^75^. For example, natural experiments, like compulsory educational reforms, that take advantage of external changes that affect EA but are unrelated to health, have shown causal effects on general health outcomes^76,77^, while twin design studies have found similarly strong causal effects on health outcomes^78^ and mortality^79^. While we were unable to assess directly which aspects of EA may be driving the identified relationship, we hypothesize aspects such as acquiring coping skills or being in a safe environment through periods of heightened suicide attempt risk (e.g., adolescence), may be important factors; however, future studies are required to elucidate specific mechanisms.

In the SVMR analysis, we found that CP reduces the risk for attempting suicide by almost 30%; however, after incorporating EA and income into a MVMR framework, CP was no longer associated with suicidal behavior, which supports previous observational literature finding that EA explains a substantial proportion of the association between CP and suicidal behavior^80–82^. CP and EA are genetically correlated^37^, and there is MR evidence supporting a bidirectional relationship between EA and CP^73^; however, using MVMR methods and sensitivity analyses to assess for possible pleiotropy, we were able to extricate their independent effects on the risk for having attempted suicide, which is critical when considering policies aimed at preventing suicide. Further, given that it has been shown that many individuals who die by suicide are never seen by health care professionals^72^, other added benefits of increased EA include using schools as ideal settings for therapeutic interventions including dialectical and cognitive-behavioral therapies^83^.

While MR does not elucidate the pathways between environmental exposures and outcomes^48^, we included in supplementary MVMR analyses potential variables that may mediate the relationship between EA and suicide attempt (i.e., economic, health-behavioral (psychiatric disorders; feeling tense, anxious, or depressed; tobacco smoking; and alcohol consumption). For example, tobacco smoking and alcohol consumption maybe some of the many possible health-behavioral pathways linking EA and suicide attempts^84,85^. We recently reported an MR study that found evidence EA causally impacts drinking patterns and the risk for alcohol dependence (AD) with increased EA reducing the frequency of binge drinking six or more drinks per occasion, and the risk for AD^39^; in this study, the effect of EA remained significant after accounting for tobacco smoking, alcohol consumption, and other associated psychiatric risk factors, suggesting prevention strategies aimed at improving EA may have a direct effect at reducing the risk for a suicide attempt. Differences in the prevalence of risk factors like alcohol consumption likely work in combination with, for example, limitations in access and differences in the ability to benefit from health care and medical information to contribute to the widening educational gradient in mortality^14,86,87^.

In addition, our polygenic instrument for EA may reflect both a biological mechanism (encoding characteristics that may increase EA) and social history (people from families with high EA may also have similar genetics)^88^ and as suicidal behavior is highly familial, and may be transmitted independently from psychiatric disorders^89^, it is possible an EA-associated influence on parental behavior may affect the child’s early developmental environments that may impact suicidal behavior later in life. While we were unable to evaluate the effect of familial EA on offspring suicide behavior because the summary-level data is not available, it has been shown by comparing polygenic scores for EA that maternal EA-linked genetics predict the child’s attainment better than the child’s own EA polygenic score^88^ suggesting it is plausible that the parental EA-genetics may also influence their child’s suicide propensity through environmental mechanisms.

Finally, while EA is—in addition to being influenced by environmental factors—polygenic^37,38^, the biological pathways through which the genetic variants comprising our genetic EA instrument impact EA is largely unknown^37^. However, the SNPs included in the instrument have been shown to be associated with several processes involved in early brain development including chromatin modification and transcription; post-translational regulations of gene expression, the formation of axons, dendrites, and synapses (which was also enriched in the CP instrument SNPs); and transmembrane transport of ions and solutes – including receptors for dopamine and serotonin^37^. Notably, altered synaptic plasticity, which may impact an individual’s ability to make appropriate adaptive responses to changing environmental stimuli, has been linked with suicidal behavior^90^, while dysregulated dopaminergic and serotonergic transmission have been associated with suicide ideation, attempts, and completions in several cohorts^91^, which together suggest potentially shared underlying biological mechanisms through which the genes associated with increased EA may impact suicide behavior.

## Strengths and limitations

There are notable strengths of the current study, including the use of multiple MR sensitivity analyses (Egger, weighted median, and weighted mode MR) that each accommodates different assumptions about genetic pleiotropy to test the robustness of the IVW estimate^92^. In addition, the GWAS summary statistics of suicide attempt risk employed as our outcome are based on hospital records, which, unlike self-reported suicidal behavior, are not likely to be prone to social-desirability and recall biases^93^. Similarly, while 90% of those attempting suicide have at least one psychiatric disorder^32^, our analyses included examining the relationship between EA and suicide attempt using models accounting for DSM-IV psychiatric disorders^49^, and additional MVMR analyses accounting for MDD, schizophrenia, and psychotic experiences, symptoms of depression, anxiety, and other adverse behaviors (alcohol consumption and smoking) potentially involved in the EA–suicide relationship, which both act a sensitivity analyses and improves the generalizability of the main findings.

Interpreting the results of this study requires an understanding of its limitations. First, we would like to emphasize the included datasets were of white individuals of primarily European ancestry, and therefore it is important to note that these results may not generalize to other ethnic and racial populations. For example, Bridge et al.^94^ found strong evidence for a racial disparity in childhood suicide rates with black children two-times more likely to commit suicide than white children. It was also recently shown that EA protected non-Hispanic Whites, but not non-Hispanic Blacks against future suicide attempts and deaths^95^. Therefore, it will be necessary to examine the EA–suicide relationship in relationship in populations of different ethnicities. In addition, participants in the UK Biobank have been shown to be more educated, lead healthier lifestyles, and have fewer health problems than the general UK population^96^. Further, the nature of suicidality makes studying suicidal behavior difficult^97^, and while suicide attempts are strong predictors of later suicide completions—25% of those dying by suicide have a failed attempt within the year prior to completion^3^—we were unable to differentiate between violent and non-violent suicide attempts, which confer different risks for later completions^98,99^.

The underlying biological mechanisms through which the instrument SNPs impact EA and the other included variables remain largely unknown; however, some of the variants are located in genomic regions regulating brain development and expressed in the neural tissue and the central nervous system throughout development and the lifespan^35^. GWS (*P*-value < 5 × 10^−8^) SNPs for each included trait explains only a small proportion of its total variance, and while our final GWS EA and CP SNPs instruments explained ~1.7% and 2.2% of the variance in EA and CP, respectively (Supplementary Table 10), it may be overly simplistic to interpret these SNPs as representations of “EA genes” or “CP genes” due to the complex interaction with environmental factors^35^.

Further, it is well documented that differences in EA are associated with more access and utilization of health care, prevention, and treatment^14,86,87^, which given the strong correlation between suicide rates with indicators of health care access^100^ suggests a potential mechanism through which EA may affect suicidal behavior. However, EA retained a robust, potentially causal, relationship with suicide attempt notwithstanding the inclusion of genetic instruments accounting for having seen a GP or psychiatrist for symptoms of “nerves, tension, anxiety, or depression.”

While MVMR enables the simultaneous assessment of the effects of two or more exposures, other horizontal pleiotropy pathways, like personality traits, may still bias the estimates^42^. However, using different sensitivity analyses that have orthogonal assumptions to test for potential pleiotropy^62^, we found similar estimates across the methods. Next, it has been shown that genetic variants associated with EA are also linked with family background and parental EA^101^_,_ suggesting that residual population stratification, assortative mating, and dynastic effects—where parental EA and CP affect the life outcomes of their offspring—could potentially explain these findings. Also, our EA instrument only evaluated the number of years of schooling at academic institutions, so resolving which aspects of education, or even how skills and values learned outside of formal academic training, impact suicide attempt risk will need to be evaluated by future studies. Similarly, CP represents an amalgamation of functions responsible for perception, thought, action, and emotion^45,102^, which might explain different aspects of the progression from suicidal ideation to attempts to completion^80^. Therefore, given the complexity of CP^38^ and the possible differential effects of various components of CP have on suicide risk, the verbal-numerical attainment and neuropsychological tests used to generate the CP outcome in the Lee et al. GWAS is insufficient to elucidate these differences. Therefore, future studies disentangling the role of distinct aspects of CP in suicidal behavior are necessary.

Further still, we were unable to distinguish the effects of gender as gender-specific suicide attempt GWASs are not yet available. However, gender is a well-known risk factor for suicide with females both demonstrating higher rates of non-fatal suicidal behavior (i.e., suicide ideation) and attempt suicide more frequently than males^103^. Conversely, males have been shown to have a higher rate of suicide completions^104^ and to use more violent and lethal methods^105^. It has been suggested that traditional gender roles may explain, in part, the observed gender differences in suicidal behavior^106^. For example, gender roles may reduce the likelihood of males to seek help for suicidal ideation or depression^106^, especially during times of economic stress where traditional gender roles may be impacted by societal expectations for males to provide for themselves and their family^106^. Similarly, while females are more likely to maintain social and family connections that may provide needed support during times of increased suicide risk^107^, the stigma associated with infertility or having children outside of marriage have been linked with increased suicidal behavior among women^107^. Since the summary association data used in this study included gender and age as covariates in the GWAS models, the outcome phenotype would be interpreted as risk of suicide attempt controlling for gender; however, future MR studies should investigate the EA–SA relationship when sex-specific suicide GWAS data becomes available.

Finally, the iPSYCH GWASs in the sample was constructed from a cohort of 1,472,762 singletons born in Denmark between 1981 and 2005, which resulted in an age-related breakdown of the 6024 included cases were as follows: 15–19 years (17.9%), 20–24 years (37.2%), 25–29 years (35.6%), and 30–34 years (9.4%)^49^. Suicidal behavior—and its predominant risk factors—is not constant over the life course^108,109^ and since the oldest iPSYCH participants were aged 34 years, we were unable to assess possible temporal components to our findings. In addition, we were unable to assess suicidal behavior within the ideation-to-action framework, where suicidal ideation and the progression from ideation to attempts are postulated to be distinct phenomena with unique predictors^97^, which makes future MR studies using GWASs that differentiate both have extended suicide behavior across the life course and differentiate between ideation and attempts necessary when the data becomes available.

## Conclusion

Our analyses align with previous observational studies which suggest that EA causally diminishes the risk for suicide attempts among white individuals of European ancestry, while the negative effect of CP on the risk of suicide attempts may be mediated through the effect of CP on EA. In conjunction with the growing number of MVMR studies to identify a direct effect of EA after accounting for CP on various physical and psychiatric outcomes, our findings suggest that targeted prevention programs that increase EA may be useful to reduce the mortality due to suicide; however, future MR studies examining the EA–suicide relationship in other racial and ethnic populations are necessary.

## Supplementary information

Supplementary Tables

## Data Availability

All analyses were conducted using publicly available data. Summary genetic data for educational attainment and cognitive performance are available from the Social Science Genetic Association Consortium portal [https://www.thessgac.org/data]. Summary genetic data for alcohol consumption are available also from the Social Science Genetic Association Consortium portal [https://www.thessgac.org/data]. Summary genetic data for household income, ever smoker status, and ever seen a general practitioner or psychiatrist (MRC-IEU UK Biobank GWAS Pipeline) and also schizophrenia, bipolar disorder, psychotic experiences, and major depressive disorder (probable) are available through the IEU Open GWAS Project [https://gwas.mrcieu.ac.uk]. Summary genetic data for suicide attempt risk are available from The Lundbeck Foundation Initiative for Integrative Psychiatric Research (iPSYCH) [https://ipsych.dk/forskning/downloads/].

## Code availability

The analysis code in R is available upon request and all data displayed in the figures are available in the Supplementary tables.

## References

[1] Collaborators GMaCoD. (2016). Global, regional, and national life expectancy, all-cause mortality, and cause-specific mortality for 249 causes of death, 1980-2015: a systematic analysis for the Global Burden of Disease Study 2015. Lancet.

[2] Hedegaard H, Curtin SC, Warner M (2018). Suicide Mortality in the United States 1999-2017. NCHS Data Brief..

[3] Owens D, Horrocks J, House A (2002). Fatal and non-fatal repetition of self-harm: systematic review. Br. J. Psychiatry.

[4] Curtis Florence TS,, Haegerich T, Luo F, Zhou C (2015). Estimated lifetime medical and work-loss costs of fatal injuries — United States, 2013. Centers for Disease Control and Prevention. MMWR Morb. Mortal. Wkly. Rep.

[5] Ludwig B, Roy B, Wang Q, Birur B, Dwivedi Y (2017). The life span model of suicide and its neurobiological foundation. Front Neurosci..

[6] Ports KA (2017). Adverse childhood experiences and suicide risk: toward comprehensive prevention. Am. J. Prev. Med..

[7] Olfson M (2017). National trends in suicide attempts among adults in the United States. JAMA Psychiatry.

[8] Clarke MC (2014). The impact of adolescent cannabis use, mood disorder and lack of education on attempted suicide in young adulthood. World Psychiatry.

[9] Dalgard OS, Mykletun A, Rognerud M, Johansen R, Zahl PH (2007). Education, sense of mastery and mental health: results from a nation wide health monitoring study in Norway. BMC Psychiatry.

[10] Drum DJ, Denmark AB (2012). Campus suicide prevention: bridging paradigms and forging partnerships. Harv. Rev. Psychiatry.

[11] Mortier P (2015). The impact of lifetime suicidality on academic performance in college freshmen. J. Affect Disord..

[12] Crosby AE, Ortega L, Stevens MR (2013). Suicides - United States, 2005-2009.. MMWR Suppl..

[13] Lorant V, Kunst AE, Huisman M, Costa G, Mackenbach J (2005). Socio-economic inequalities in suicide: a European comparative study. Br. J. Psychiatry.

[14] Phillips JA, Hempstead K (2017). Differences in U.S. suicide rates by educational attainment, 2000-2014. Am. J. Prev. Med..

[15] Abdel-Rahman O (2019). Socioeconomic predictors of suicide risk among cancer patients in the United States: a population-based study. Cancer Epidemiol..

[16] Olshansky SJ (2012). Differences in life expectancy due to race and educational differences are widening, and many may not catch up. Health Aff..

[17] Deary IJ, Strand S, Smith P, Fernandes C (2007). Intelligence and educational achievement. Intelligence.

[18] Deary IJ, Penke L, Johnson W (2010). The neuroscience of human intelligence differences. Nat. Rev. Neurosci..

[19] Gorlyn M (2015). Treatment-related improvement in neuropsychological functioning in suicidal depressed patients: paroxetine vs. bupropion. Psychiatry Res..

[20] Dombrovski AY (2008). Cognitive performance in suicidal depressed elderly: preliminary report. Am. J. Geriatr. Psychiatry.

[21] Westheide J (2008). Executive performance of depressed suicide attempters: the role of suicidal ideation. Eur. Arch. Psychiatry Clin. Neurosci..

[22] Leamer EE (1983). Let’s take the con out of econometrics. Am. Econ. Rev..

[23] Smith GD, Ebrahim S (2003). ‘Mendelian randomization’: can genetic epidemiology contribute to understanding environmental determinants of disease?. Int. J. Epidemiol..

[24] Smith GD, Ebrahim S (2001). Epidemiology - is it time to call it a day?. Int. J. Epidemiol..

[25] Phillips AN, Smith GD (1991). How independent are independent effects - relative risk-estimation when correlated exposures are measured imprecisely. J. Clin. Epidemiol..

[26] Westman J, Hasselstrom J, Johansson SE, Sundquist J (2003). The influences of place of birth and socioeconomic factors on attempted suicide in a defined population of 4.5 million people. Arch. Gen. Psychiatry.

[27] Qin P, Agerbo E, Mortensen PB (2003). Suicide risk in relation to socioeconomic, demographic, psychiatric, and familial factors: a national register-based study of all suicides in Denmark, 1981-1997. Am. J. Psychiatry.

[28] Bothwell LE, Greene JA, Podolsky SH, Jones DS (2016). Assessing the gold standard — lessons from the history of RCTs. N. Engl. J. Med..

[29] Davey Smith G, Hemani G (2014). Mendelian randomization: genetic anchors for causal inference in epidemiological studies. Hum. Mol. Genet..

[30] Smith GD (2011). Use of genetic markers and gene-diet interactions for interrogating population-level causal influences of diet on health. Genes Nutr..

[31] Mirkovic B (2016). Genetic association studies of suicidal behavior: a review of the past 10 years, progress, limitations, and future directions. Front. Psychiatry.

[32] Mullins N (2019). GWAS of suicide attempt in psychiatric disorders and association with major depression polygenic risk scores. Am. J. Psychiatry.

[33] Strawbridge RJ (2019). Identification of novel genome-wide associations for suicidality in UK Biobank, genetic correlation with psychiatric disorders and polygenic association with completed suicide. EBioMedicine.

[34] Harrison, R., Munafo, M. R., Davey Smith, G. & Wootton, R. E. Examining the effect of smoking on suicidal ideation and attempts: a triangulation of epidemiological approaches. The British Journal of Psychiatry 2020: 1–7. 10.1192/bjp.2020.68 PMC7705667 32290872

[35] Okbay A (2016). Genome-wide association study identifies 74 loci associated with educational attainment. Nature.

[36] Kramer AF, Bherer L, Colcombe SJ, Dong W, Greenough WT (2004). Environmental influences on cognitive and brain plasticity during aging. J. Gerontol..

[37] Lee JJ (2018). Gene discovery and polygenic prediction from a genome-wide association study of educational attainment in 1.1 million individuals. Nat. Genet..

[38] Trampush JW (2017). GWAS meta-analysis reveals novel loci and genetic correlates for general cognitive function: a report from the COGENT consortium. Mol. Psychiatry.

[39] Rosoff, D. B. et al. Educational attainment impacts drinking behaviors and risk for alcohol dependence: results from a two-sample Mendelian randomization study with ~780,000 participants. *Mol. Psychiatry*10.1038/s41380-019-0535-9 (2019). 10.1038/s41380-019-0535-9 PMC7182503 31649322

[40] Gage SH, Bowden J, Smith GD, Munafo MR (2018). Investigating causality in associations between education and smoking: a two-sample Mendelian randomization study. Int. J. Epidemiol..

[41] Sanderson E, Davey Smith G, Windmeijer F, Bowden J (2019). An examination of multivariable Mendelian randomization in the single-sample and two-sample summary data settings. Int J. Epidemiol..

[42] Davies NM (2019). Multivariable two-sample Mendelian randomization estimates of the effects of intelligence and education on health. Elife.

[43] Carter AR (2019). Understanding the consequences of education inequality on cardiovascular disease: mendelian randomisation study. BMJ.

[44] Tillmann T (2017). Education and coronary heart disease: mendelian randomisation study. BMJ..

[45] Gill D, Efstathiadou A, Cawood K, Tzoulaki I, Dehghan A (2019). Education protects against coronary heart disease and stroke independently of cognitive function: evidence from Mendelian randomization. Int. J. Epidemiol..

[46] Burgess S, Thompson SG (2015). Multivariable Mendelian randomization: the use of pleiotropic genetic variants to estimate causal effects. Am. J. Epidemiol..

[47] Richardson TG (2020). Evaluating the relationship between circulating lipoprotein lipids and apolipoproteins with risk of coronary heart disease: a multivariable Mendelian randomisation analysis. PLoS Med..

[48] Sanderson E, Davey Smith G, Bowden J, Munafò MR (2019). Mendelian randomisation analysis of the effect of educational attainment and cognitive ability on smoking behaviour. Nat. Commun..

[49] Erlangsen A (2018). Genetics of suicide attempts in individuals with and without mental disorders: a population-based genome-wide association study. Mol Psychiatry..

[50] Morris TT, Davies NM, Davey Smith G (2020). Can education be personalised using pupils’ genetic data?. Elife.

[51] Hill WD (2019). A combined analysis of genetically correlated traits identifies 187 loci and a role for neurogenesis and myelination in intelligence. Mol. Psychiatry.

[52] Elsworth, B. et al. *MRC IEU UK Biobank GWAS Pipeline Version 1.*10.5523/bris.2fahpksont1zi26xosyamqo8rr (2017). Retrieved Feb 2 2019.

[53] Clarke TK (2017). Genome-wide association study of alcohol consumption and genetic overlap with other health-related traits in UK Biobank (N=112 117). Mol. Psychiatry.

[54] Legge SE (2019). Association of genetic liability to psychotic experiences with neuropsychotic disorders and traits. JAMA Psychiatry.

[55] Howard DM (2018). Genome-wide association study of depression phenotypes in UK Biobank identifies variants in excitatory synaptic pathways. Nat. Commun..

[56] Ripke S (2014). Biological insights from 108 schizophrenia-associated genetic loci. Nature.

[57] Hou L (2016). Genome-wide association study of 40,000 individuals identifies two novel loci associated with bipolar disorder. Hum. Mol. Genet..

[58] Aschard H, Vilhjálmsson BJ, Joshi AD, Price AL, Kraft P (2015). Adjusting for heritable covariates can bias effect estimates in genome-wide association studies. Am. J. Hum. Genet..

[59] Palmer TM (2012). Using multiple genetic variants as instrumental variables for modifiable risk factors. Stat. Methods Med. Res..

[60] Sanderson, E., Spiller, W. & Bowden, J. Testing and correcting for weak and pleiotropic instruments in two-sample multivariable mendelian randomisation. Preprint at https://www.biorxiv.org/content/10.1101/2020.04.02.021980v1. 10.1002/sim.9133 PMC9479726 34338327

[61] Burgess S, Davies NM, Thompson SG (2016). Bias due to participant overlap in two-sample Mendelian randomization. Genet. Epidemiol..

[62] Hemani G (2018). The MR-Base platform supports systematic causal inference across the human phenome. Elife.

[63] Bowden J, Smith GD, Burgess S (2015). Mendelian randomization with invalid instruments: effect estimation and bias detection through Egger regression. Int. J. Epidemiol..

[64] Davey Smith G (2016). Assessing the suitability of summary data for two-sample Mendelian randomization analyses using MR-Egger regression: the role of the I2 statistic. Int. J. Epidemiol..

[65] Bowden J, Davey, Smith G, Haycock PC, Burgess S (2016). Consistent estimation in Mendelian randomization with some invalid instruments using a weighted median estimator. Genet. Epidemiol..

[66] Hartwig FP, Davey Smith G, Bowden J (2017). Robust inference in summary data Mendelian randomization via the zero modal pleiotropy assumption. Int. J. Epidemiol..

[67] Rees JMB, Wood AM, Burgess S (2017). Extending the MR-Egger method for multivariable Mendelian randomization to correct for both measured and unmeasured pleiotropy. Stat. Med..

[68] Bowden J (2017). A framework for the investigation of pleiotropy in two-sample summary data Mendelian randomization. Stat. Med..

[69] Bowden J (2019). Improving the accuracy of two-sample summary-data Mendelian randomization: moving beyond the NOME assumption. Int J. Epidemiol..

[70] Verbanck M, Chen CY, Neale B, Do R (2018). Detection of widespread horizontal pleiotropy in causal relationships inferred from Mendelian randomization between complex traits and diseases. Nat. Genet..

[71] Amrhein V, Greenland S, McShane B (2019). Scientists rise up against statistical significance. Nature.

[72] Bostwick JM, Pabbati C, Geske JR, McKean AJ (2016). Suicide attempt as a risk factor for completed suicide: even more lethal than we knew. Am. J. Psychiatry.

[73] Anderson, E. L. et al. Education, intelligence and Alzheimer’s disease: Evidence from a multivariable two-sample Mendelian randomization study. *Int. J. Epidemiol*. 10.1093/ije/dyz280 (2020). 10.1093/ije/dyz280 PMC7660137 32003800

[74] Case A, Deaton A (2015). Rising morbidity and mortality in midlife among white non-Hispanic Americans in the 21st century. Proc. Natl Acad. Sci. USA.

[75] Zajacova A, Lawrence EM (2018). The relationship between education and health: reducing disparities through a contextual approach. Annu Rev. Public Health.

[76] Fletcher JM (2015). New evidence of the effects of education on health in the US: compulsory schooling laws revisited. Soc. Sci. Med..

[77] Davies NM, Dickson M, Smith GD, van den Berg GJ, Windmeijer F (2018). The causal effects of education on health outcomes in the UK Biobank. Nat. Hum. Behav..

[78] Gerdtham UG, Lundborg P, Lyttkens CH, Nystedt P (2016). Do education and income really explain inequalities in health? Applying a twin design. Scand. J. Econ..

[79] Lundborg P, Lyttkens CH, Nystedt P (2016). The effect of schooling on mortality: new evidence from 50,000 Swedish twins. Demography.

[80] Hansson Bittár N, Falkstedt D, Sörberg Wallin A (2020). How intelligence and emotional control are related to suicidal behavior across the life course – A register-based study with 38-year follow-up. Psychol. Med..

[81] Sorberg A, Allebeck P, Melin B, Gunnell D, Hemmingsson T (2013). Cognitive ability in early adulthood is associated with later suicide and suicide attempt: the role of risk factors over the life course. Psychol. Med..

[82] Sorberg Wallin A, Allebeck P, Gustafsson JE, Hemmingsson T (2018). Childhood IQ and mortality during 53 years’ follow-up of Swedish men and women. J. Epidemiol. Community Health.

[83] Evans R, Hurrell C (2016). The role of schools in children and young people’s self-harm and suicide: systematic review and meta-ethnography of qualitative research. BMC Public Health.

[84] Pompili M (2010). Suicidal behavior and alcohol abuse. Int. J. Environ. Res. Public Health.

[85] Poorolajal J, Darvishi N (2016). Smoking and suicide: a meta-analysis. PLoS ONE.

[86] Lleras-Muney A (2005). The relationship between education and adult mortality in the United States. Rev. Econ. Stud..

[87] Meara ER, Richards S, Cutler DM (2008). The gap gets bigger: changes in mortality and life expectancy, by education, 1981-2000. Health Aff..

[88] Belsky DW (2018). Genetic analysis of social-class mobility in five longitudinal studies. Proc. Natl Acad. Sci..

[89] Brent DA, Melhem N (2008). Familial transmission of suicidal behavior. Psychiatr. Clin. North Am..

[90] Fossati P, Radtchenko A, Boyer P (2004). Neuroplasticity: from MRI to depressive symptoms. Eur. Neuropsychopharmacol..

[91] Carballo JJ, Akamnonu CP, Oquendo MA (2008). Neurobiology of suicidal behavior. An integration of biological and clinical findings. Arch. Suicide Res..

[92] Lawlor DA (2016). Commentary: two-sample Mendelian randomization: opportunities and challenges. Int. J. Epidemiol..

[93] Choi BC, Pak AW (2005). A catalog of biases in questionnaires. Prev. Chronic Dis..

[94] Bridge JA (2018). Age-related racial disparity in suicide rates among US youths from 2001 through 2015. JAMA Pediatrics..

[95] Assari S (2019). Higher educational attainment is associated with lower risk of a future suicide attempt among non-Hispanic Whites but not non-Hispanic Blacks. J. Racial Ethn. Health Disparities..

[96] Fry A (2017). Comparison of sociodemographic and health-related characteristics of UK biobank participants with those of the general population. Am. J. Epidemiol..

[97] Klonsky ED, May AM, Saffer BY (2016). Suicide, suicide attempts, and suicidal ideation. Annu Rev. Clin. Psychol..

[98] Runeson B, Tidemalm D, Dahlin M, Lichtenstein P, Långström N (2010). Method of attempted suicide as predictor of subsequent successful suicide: national long term cohort study. BMJ.

[99] Stefansson J, Nordstrom P, Jokinen J (2012). Suicide Intent Scale in the prediction of suicide. J. Affect Disord..

[100] Tondo L, Albert MJ, Baldessarini RJ (2006). Suicide rates in relation to health care access in the United States: an ecological study. J. Clin. Psychiatry.

[101] Kong A (2018). The nature of nurture: Effects of parental genotypes. Science.

[102] Deary IJ, Johnson W (2010). Intelligence and education: causal perceptions drive analytic processes and therefore conclusions. Int. J. Epidemiol..

[103] Krug EG, Mercy JA, Dahlberg LL, Zwi AB (2002). The world report on violence and health. Lancet.

[104] Värnik P (2012). Suicide in the world. Int J. Environ. Res. Public Health.

[105] Han B (2016). Suicidal ideation, suicide attempt, and occupations among employed adults aged 18-64years in the United States. Compr. Psychiatry.

[106] Möller-Leimkühler AM (2003). The gender gap in suicide and premature death or: why are men so vulnerable?. Eur. Arch. Psychiatry Clin. Neurosci..

[107] Girard C (1993). Age, gender, and suicide: a cross-national analysis. Am. Sociol. Rev..

[108] Hysinger EB (2011). Suicidal behavior differs among early and late adolescents treated with antidepressant agents. Pediatrics.

[109] Conwell Y (1998). Age differences in behaviors leading to completed suicide. Am. J. Geriatr. Psychiatry.

